# Structural, Thermal, and Release Properties of Hybrid Materials Based on Layered Zinc Hydroxide and Caffeic Acid

**DOI:** 10.3390/nano10010163

**Published:** 2020-01-17

**Authors:** Christhy V. Ruiz, María E. Becerra, Oscar Giraldo

**Affiliations:** 1 Laboratorio de Materiales Nanoestructurados y Funcionales, Facultad de Ciencias Exactas y Naturales, Universidad Nacional de Colombia- Sede Manizales, Kilometro 9 vía al aeropuerto, La Nubia, 170003 Manizales, Colombia; cvruizma@unal.edu.co (C.V.R.); mebecerrah@unal.edu.co (M.E.B.); 2 Grupo de Investigación en Procesos Químicos, Catalíticos y Biotecnológicos, Universidad Nacional de Colombia-Sede Manizales, Kilometro 9 vía al aeropuerto, La Nubia, 170003 Manizales, Colombia; 3 Departamento de Ingeniería Química, Facultad de Ingeniería y Arquitectura, Universidad Nacional de Colombia-Sede Manizales, Kilometro 9 vía al aeropuerto, La Nubia, 170003 Manizales, Colombia; 4 Departamento de Física y Química, Facultad de Ciencias Exactas y Naturales, Universidad Nacional de Colombia-Sede Manizales, Kilometro 9 vía al aeropuerto, La Nubia, 170003 Manizales, Colombia; 5 Departamento de Química, Universidad de Caldas, Calle 65 No. 26-10, 17001 Manizales, Colombia

**Keywords:** layered zinc hydroxide, caffeic acid, hybrid material, release systems, immobilization

## Abstract

Caffeic acid (CA) molecules were immobilized in a layered inorganic host matrix based on zinc hydroxide structures with different starting interlayer anions, nitrate, and acetate. The chemical composition, structure, thermal stability, morphology, and surface of the host matrices and hybrid compounds were analyzed by X-ray diffraction (XRD), themogravimetric/differencial thermal analysis (TG/DTA), Fourier transform infrarred spectroscopy (FT-IR), scanning electron microscopy (SEM), and X-ray photoelectron spectroscopy (XPS). Additionally, the surface charge of the materials was investigated using zeta potential at pH ~7. The results show an influence of the surface charge on the chemical, interaction, and structure of the resulting hybrid materials as a function of the starting layered structures. An expansion of the basal spacing to 10.20 Å for zinc hydroxide nitrate (ZHN), and a shrinkage to 10.37 Å for zinc hydroxide acetate (ZHA). These results suggest that the CA lies with a tilt angle in the interlayer region of the inorganic host matrix. The immobilization of CA is favored in ZHN, with respect to ZHA, because a single-layered phase was identified. A higher thermal stability at 65 °C was observed for ZHN-CA than for ZHA-CA. The evaluation of the release behavior showed a higher percentage of CA released from ZHN than ZHA, and the release mechanism was described by the Elovich model. The hybrid materials show potential characteristics for use as bioactive delivery systems.

## 1. Introduction

Layered inorganic materials, such as layered double hydroxides (LDH) and zinc hydroxy salts (ZHS), are characterized by retaining different chemical species compatible in the interlayer region due to the charge of their layers, thereby achieving the charge balance of the layered structure. This possibility enables the creation of a wide variety of hybrid materials with new applications and functionalities [1]. The differences between LDH and ZHS in terms of the layer structure and bonding confer different physicochemical and interfacial properties to the materials. The materials of the LDH family consist of octahedral layers formed by the partial substitution of divalent metal ions (M^2+^) by trivalent metal ions (M^3+^), with the interlayer anion balancing the positive charge. In contrast, in the ZHS, one-quarter of the octahedral sites of Zn^2+^ atoms are empty. Instead, Zn^2+^ ions are placed in tetrahedral sites at the bottom and top of each empty octahedron [2], with three hydroxyl groups forming the base of the tetrahedron, and, on the apex, a water molecule is coordinated. With regard to layered double hydroxides (LDH), a wide range of molecules such as complex ions, vitamins, organic acids, genes, and drugs, amongst others, were extensively investigated [3,4,5,6,7]. However, in terms of ZHS, there is still much to be explored about immobilization studies of functional molecules, such as antioxidants, dyes, and drugs [8,9].

The immobilization process, using zinc hydroxide salts (ZHS) such as zinc hydroxide nitrate (ZHN) and zinc hydroxide acetate (ZHA), takes advantage of the structural characteristics of these layered host materials and their possibility to form covalent binding with different chemical species. The zinc hydroxide salts consist of cationic zinc hydroxy layers and associated anions. The positive charge of the layer can be compensated for with free nitrate ions for ZHN and acetate anions for ZHA in the interlayer region [10,11]. The new materials using ZHN as a host matrix exhibited improved characteristics as in the case of ciprofloxacin intercalated into ZHN, where the intercalated compound showed stronger anti-cancer effects against A549 cancer cells compared to the pure ciprofloxacin molecule [12], or the nanocomposite developed by the intercalation of hippuric acid into ZHN, which presented better anti-microbial properties against drug-resistant bacteria compared to free hippuric acid [13].

Bioactive molecules, of natural origin, attracted considerable attention in the last few years due to their valuable biological and physiological properties [14]. The exchange anion capability, offered by ZHN and ZHA, also allows the incorporation of these types of biomolecules. One such bioactive molecule is caffeic acid (CA), which possesses antioxidant, anticancer, anti-inflammatory, and antiviral properties [15,16,17]. The anion exchange of caffeic acid in Mg–Al LDH was evaluated by Ito et al.; however, their study was only conducted in order to synthesize a CA–LDH intercalated compound [18]. On the other hand, Choy et al. encapsulated caffeic acid in zinc hydroxide nitrate to evaluate the ultraviolet UVA1 screening ability of the nanohybrid material. However, the thermal and surface characterization was not studied in detail [19].

Therefore, this study aims to incorporate caffeic acid into ZHN and ZHA inorganic host matrices, to determine the effect on the structural, thermal, and surface properties of the hybrid materials obtained from different layered starting materials for the immobilization process of the active molecule, as well as to evaluate the release behavior of the hybrid material for practical cosmetic and pharmaceutical applications. 

## 2. Materials and Methods

### Materials and Synthesis of Samples

#### 2.1.1. Synthesis of Layered Zinc Hydroxide

Zinc hydroxide nitrate (ZHN) and zinc hydroxide acetate (ZHA) were synthesized using the method reported in previous work [20,21]. For each compound, aqueous solutions with 23.88 g of Zn(NO_3_)_2_∙4H_2_O (Merck 98.9%, St. Louis, MO, USA) and 16.24 g of Zn(CH_3_CO_2_)_2_ (Merck 99.9%, St. Louis, MO, USA) were prepared in 60 mL of deionized water. Thereafter, the solutions were mixed in different suspensions, with 7.39 g/60 mL of zinc oxide (ZnO) (Sigma Aldrich 99%–100%, St. Louis, MO, USA) with constant stirring for 24 h. The white slurries were centrifuged, washed three times with deionized water, and dried at room temperature.

#### 2.1.2. Synthesis of Hybrid Material

The immobilization of caffeic acid (CA) (Alfa Aesar, 3,4-dihydroxycinnamic acid, predominantly *trans*, 99%, Ward Hill, MA, USA) was conducted by the dispersion of 5.00 g of ZHN and ZHA, independently, in a suspension of 50.0 mL of deionized water with 1.82 g of CA. The resultant suspensions were stirred at room temperature for 24 h. The solid product was recovered by centrifuge and washed three times with deionized water as described above. The product was dried for 72 h at room temperature.

#### 2.1.3. Characterization of Hybrid Materials

The characterization of the materials was done by elemental analysis, X-ray diffraction, thermal analysis, Fourier transform-infrared spectroscopy, scanning electron microscopy, and X-ray photoelectron spectroscopy. The chemical composition was estimated from a quantitative combustion technique of carbon, hydrogen, and nitrogen on a Leco TruSpec Micro CHNSO elemental analyzer (St. Joseph, MI, USA), and atomic absorption spectroscopy to determine zinc content on a Thermo Scientific iCE 3000 AA spectrometer (Waltham, MA, USA). The surface charge of the materials was measured using a Malvern Zetasizer Nano ZS instrument (Alcobendas, Madrid, Spain) in 0.1 mol/L NaOH. The XRD patterns were conducted with a Rigaku Miniflex II Diffractometer (Woodlands, TX, USA) with CuKα radiation (1.541 Å) at 30 kV and 15 mA. Thermal analysis was carried out using TGA Q500 (New Castle, DE, USA) from 25 °C to 700 °C at a heating rate of 10 °C∙min^−1^ in a nitrogen atmosphere. The FTIR spectra were recorded at room temperature for powder samples with a Mid-Infrared FT-IR Nicolet iS5 Thermo Fisher Spectrometer (Waltham, MA, USA) in the range of 4000 cm^−1^ to 400 cm^−1^, with a resolution of 4.0 cm^−1^ using pellets prepared with KBr. Scanning electron microscope images were collected on a JEOL JSM-6490LV equipment (North Billerica, MA, USA) with an acceleration voltage of 20 kV. X-ray photoelectron spectroscopy was performed on a PHI 5000 Versa Probe II Scanning XPS Microprobe (Chanhassen, MN, USA) with monochromatic Al Kα radiation. The spectra were obtained at a takeoff angle of 45° to the sample normal using a spot diameter of 100 µm. In all samples, the binding energy was referenced using the C 1*s* peak at 284.5 eV [22]. The high-resolution spectra were used for elemental quantification of the compounds using Multipak data processing software [23]. The schematic representation of the intercalation process in this work was made with CrystalMaker software (Begbroke, OX5, UK) [24].

#### 2.1.4. Controlled Release Studies

The experiments were performed using a buffer solution (pH 7.1) by dissolving NaH_2_PO_4_ (0.0534 g) and Na_2_HPO_4_ (0.2195 g) in deionized water to make 1000 mL of a solution. The hybrid compounds (0.050 g) were added to 1000 mL of the buffer solution with continuous stirring. Aliquots of 5.0 mL were collected from the reaction at regular intervals. The concentration in each one of the aliquots was measured in triplicate by UV spectroscopy using a lambda UV–visible light spectrophotometer at 19.0 ± 1 °C. The maximum absorption wavelength in all solutions of the hybrid materials, in the release study, was 265 nm. Initially, the calibration curve was generated to calculate the amount of intercalated CA using eight aqueous solutions with concentrations ranging from 0.0 to 50.0 mg/L of phosphate buffer solution (pH 7.1) with 50.0 mg/L of CA concentration. The least-square method was employed to fit the equation of the calibration curve of the *y* = *ax* + *b* form, resulting in *a* = 4.123 × 10^−2^, and *b* = −18.51 × 10^−3^ with *R*^2^ = 0.999, where *x* is the absorbance and *y* is the CA solution concentration, expressed in mg/L.

## 3. Results and Discussion

### 3.1. Elemental Analysis

The elemental analyses of the hybrid materials based on layered zinc hydroxide salts and caffeic acid, as well as those of the inorganic host matrices, are presented in Table 1. The calculated formulas were estimated from the stoichiometry comparison of the experimental data and theoretical values of the ideal formula Zn_5_(OH)_8_(CA)_2_∙2H_2_O. The absence of the starting interlayer anions of the host matrices (nitrate and acetate anions), taking into account the calculated formula from the chemical composition, indicates that the guest anion (caffeate) was immobilized into the layered structure to form the hybrid materials. For the hybrid material from zinc hydroxide nitrate, a content of 43.6% of caffeic acid was determined, whereas, for zinc hydroxide acetate, the content was 43.1%. These estimated values are close to that represented by the theoretical formula. The difference in the composition of the hybrid materials as a function of the starting host matrices, zinc hydroxide nitrate (ZHN) and zinc hydroxide acetate (ZHA), given the variation of the content of water, hydroxyl groups, and caffeate anions, can be related to the difference in the interaction mode between the guest anion and the layers after displacement of the starting anions, which led to a modification of structure for the resulting hybrid material. 

### 3.2. Interfacial Characterization

**Figure 1:**
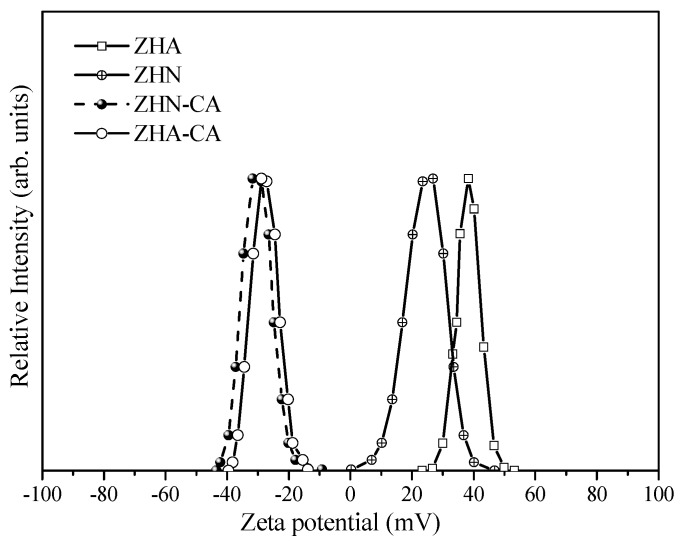
Zeta potential distribution of ZHN, ZHA, ZHN–CA, and ZHA–CA materials.

The surface charges for the inorganic host matrices, zinc hydroxide nitrate and zinc hydroxide acetate (ZHN and ZHA), as well as the hybrid materials, were studied by measuring the zeta potential in an aqueous media of pH 7.0. Zeta potentials of both inorganic host matrices in the suspension were positive, with an average zeta potential given by the instrument of 24.2 ± 1.9 mV for ZHN and 38.4 ± 1.8 mV for ZHA. A zeta potential distribution range from 23 to 53 mV for ZHA and from 0.2 to 43 mV for ZHN can be estimated in Figure 1. As it was reported by Xu et al. and Mahmoud et al., the zeta potential for LDH materials (Mg–Al LDH) has a positive value. This value is mainly related to the structural positive charge on the electric double layer on the LDH surface [25]. In a similar case, for layered zinc hydroxide salts, the positive values can be attributed to the structural positive charge given by the Zn^2+^ ions placed in the octahedral and tetrahedral sites forming the layers [2]. However, the interior structural charge of the interlayer region is screened by the interlayer anions. The charge balance of the layers with the interlayer anions is not complete in the layered structure since the surface-adsorbed anions thermally vibrate and temporarily leave the electric double layer (Stern layer) toward the other electric layer called the diffusion layer, where static anions can always be found, which leads to the layered particles associated with the Stern layer having a positive charge [25].

Therefore, the difference in the zeta potential value between the layered host matrices can be influenced by the particular characteristics of interaction between the layers and the interlayer anion in the aqueous medium. Therefore, in the case of the ZHN, given its chemical composition from the elemental analysis, a strong adsorption of the CO_3_^2−^ and NO_3_^−^ anions on the surface of the positive layers of the ZHN occurred to form the Stern layer (negative charge), which induced a lower amount of the anions present in the diffusion layer, giving rise to a smaller zeta potential value. A different response was observed for ZHA, in which a more significant zeta potential value was obtained. This response is likely associated with an electrostatic interaction through the hydrogen bonds between the water molecule located on the apex of the tetrahedral units of the layers and the oxygen atom of the acetate anion, which resulted in more acetate anions being found on the diffusion layer of the aqueous medium. Also, the difference in the composition chemical of the hydroxyl layers regarding ZHN can affect the results of the zeta potential value. The values of zeta potential are similar to those for LDH with CO_3_^2−^ anions reported by Xu et al. [26] and Tran et al. [27].

The values of the zeta potential of the hybrid materials based on caffeic acid and both layered zinc hydroxide host matrices were found to be negative in that ae pH of ~7.0. The average zeta potential for ZHN–CA was −29.0 ± 0.3 mV, and ZHA–CA had a value of −28.7 ± 0.2 mV, with distribution zeta potentials in the range of −9.2 to −43.5 mV and −13.8 to 39.5 mV for each hybrid material, respectively. The negative surface charge for the hybrid material is a fact that demonstrates the immobilization of the organic anion into the layered structure. With the pH in the aqueous medium, the H+ ions of the functional groups of the caffeic acid (carboxylate group) are liberated, which leads to a more negative value of zeta potential [28]. The strong electrostatic attraction between the oxygen atom of the carboxylate group of the caffeate anion and the Zn^2+^ atoms of the tetrahedral units can be the cause of the zeta potential response. As a consequence of the surface charge of the starting layered host matrix, as well as the resulting chemical composition of the hybrid materials, a change in the values of zeta potential (ZHN–CA and ZHA–CA) was obtained. Based on the results of the zeta potential values, the interfacial behavior indicates suspension stability based on electrostatic interactions of the hybrid materials and the starting host matrices, which have differences owing to the chemical composition and interactions [2,25,29,30].

### 3.3. Powder X-Ray Diffraction (PXRD)

**Figure 2:**
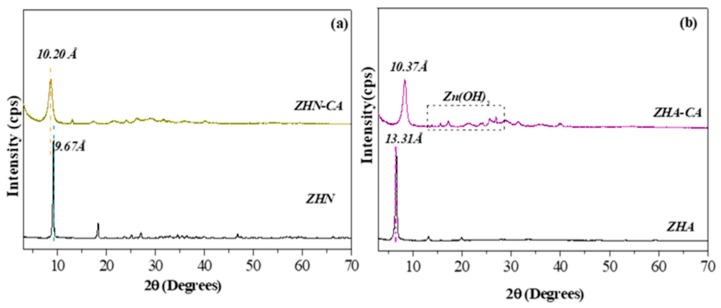
Powder X-Ray Diffraction patterns of (**a**) starting material and hybrid materials based on ZHN and (**b**) inorganic host matrix ZHA, and their intercalated compound with caffeinate anion.

The XRD patterns of the starting inorganic matrices and hybrid materials are shown in Figure 2. From now on, the inorganic–organic hybrid materials are referred to as ZHN–CA and ZHA–CA. The X-ray pattern of the inorganic matrix and hybrid materials from ZHN are shown in Figure 2a. For the host matrix ZHN, the intense reflection was identified as the phase Zn_5_(OH)_8_(NO_3_)_2_∙2H_2_O (JCDS 72-0627) with a *d*-value of around 9.67 Å [19,21]. The XRD pattern for ZHN–CA revealed a shift toward lower angles of reflection, which indicates an expansion of the interlayer space of the layered structure. The increase in *d*-value from 9.67 Å to 10.20 Å revealed the incorporation of a larger molecule than that of the starting anions in the interlayer region. Unlike the results obtained for the ZHN inorganic matrix, the results from the hybrid material with ZHA as the starting material (Figure 2b) revealed a reduction of the interlayer space from 13.31 Å [20] to 10.37 Å for ZHA–CA, which is yet to be reported to our knowledge. These results are in accordance with those reported by Choy et al. [19], who recorded a *d*-spacing of 10.44 Å for intercalated CA in a layered zinc hydroxide nitrate matrix (CA–ZBS). The decomposition of this ZHA–CA hybrid compound was evident, in comparison with the results obtained for hybrid material from ZHN inorganic host matrix, since reflections associated with Zn(OH)_2_ were observed. Furthermore, the evidence of this mixture of phases in the ZHA–CA hybrid material might justify the differences between the percentages of the elemental content observed and calculated.

Particularly, for both starting materials, the predominant arrangement, taking into account the *d*-value, is that where the organic anions (caffeate) are arranged with a tilt angle close to 70° along the *z*-axis inside the interlayer space of the layered inorganic host matrix. This orientation possibly creates stronger interactions between the carboxylate group, present in the caffeic acid molecule, and the Zn^2+^ atoms of the tetrahedral units, which favor the substitution of the water molecules in the apex of tetrahedron of the layers in both cases and result in the coordination of the guest anion to the layers [31].

The differences in the *d*-values of the resulting hybrid material, as a function of the inorganic host matrix, can be related to the initial chemical composition and surface charge of the starting layered structure. The more significant surface charge of the layers into ZHA can lead to a stronger interaction with the guest anions, giving rise to the shrinkage of the resulting layered hybrid structure, ZHA–CA, and the difference in chemical composition can affect the degradation of the layers for this hybrid material with respect to ZHN–CA.

**Figure 3:**
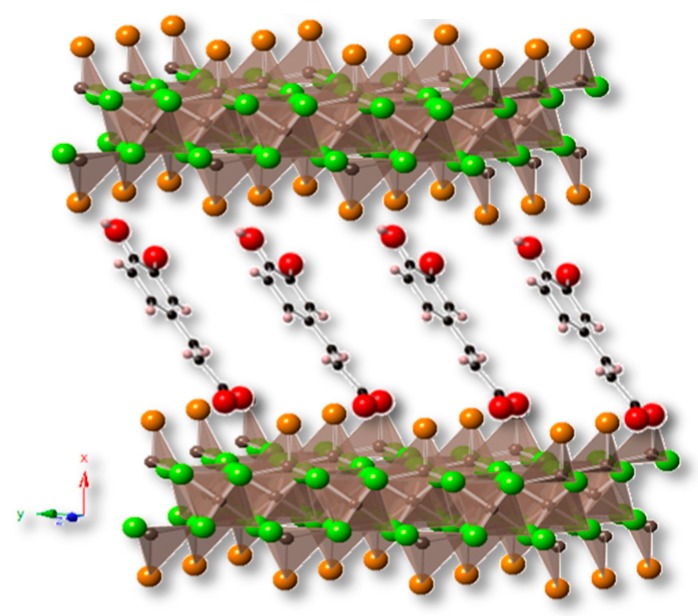
Representation of the caffeate anion arrangement in the interlayer space of layered structure based on ZHA and ZHN host matrix.

A schematic representation of the arrangement of the caffeate anions in the interlayer space of the host matrices is illustrated in Figure 3.

### 3.4. Thermal Analysis (TG/DTA)

**Figure 4:**
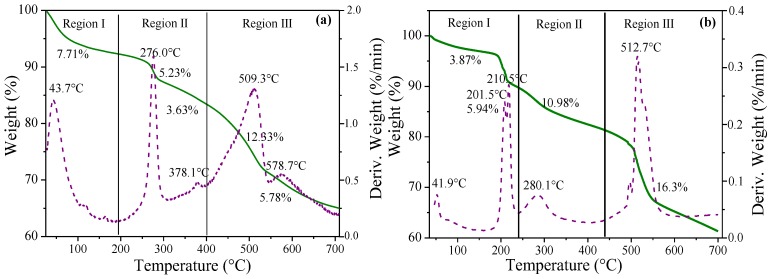
Thermogravimetric analysis (TG/DTA) of the hybrid compounds for (**a**) ZHN–CA and (**b**) ZHA–CA.

The TG/DTA curves are shown in Figure 4a,b. The thermal decomposition of ZHN–CA occurred in three regions with a total mass loss of 34.68%. The first region (I) was identified around 43.71 °C with a loss of 7.71%. This region corresponds to the removal of physiosorbed water in the interlayer region. In the second region (II), a mass loss of 5.23% and 3.63%, with a sharp inflection points at 276.0 and 378.1 °C in the derivative analysis, corresponds to the dehydroxylation process of the layers. In the third region (III), two peaks were observed, one at 509.3 °C with a mass loss of 12.33%, attributed to the quantitative decomposition of organic material, and a weak peak at 578.7 °C with a weight loss of 5.78%, corresponding to the decomposition of the residue of the immobilized organic anions [32].

For ZHA–CA, the thermal decomposition revealed that the total mass loss value was 37.09% upon heating at 700 °C, which was slightly higher than that of ZHN–CA. In the first region (I), a mass loss of 3.87% is associated with the removal of physiosorbed water molecules [19]. The peaks at 201.5, 210.5, and 280.1 °C, in the derivative analysis, are associated with the beginning of the dehydroxylation process, as well as the decomposition of the organic species, which is evidenced with a mass loss of 16.92%. The third region had a sharp peak with a mass loss of 16.30% at 512.7 °C, which can be associated with the decomposition of the immobilized organic species (CA). From the results, the hybrid material ZHN–CA showed a thermal stability of 65.5 °C higher than that of the ZHA–CA, since the start of the dehydroxylation process was delayed.

### 3.5. FT-IR Spectroscopy

**Figure 5:**
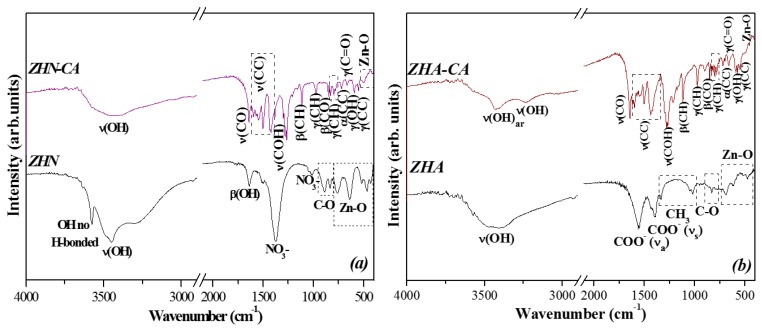
Fourier transform infrared spectra for: (**a**) ZHN and ZHN–CA, and (**b**) ZHA and ZHA–CA.

The FT-IR spectra of the host materials ZHN and ZHA, and hybrid compounds, ZHN–CA and ZHA–CA, are shown in Figure 5a,b. For the host matrix, ZHN, a broad absorption band around 3448 cm^−1^ attributed to the O–H stretching, due to the presence of hydroxyl groups of ZHN and physically adsorbed water, can be observed. The sharp band at 3571 cm^−1^ can be associated with OH stretching vibrations, non-H-bonded [33,34,35]. The band at 1638 cm^−1^ can be attributed to the OH bending vibrational mode of water molecules occupying the interlayer spacing [35,36]. The strong band located at 1378 cm^−1^ corresponds to the stretching vibration of free nitrate groups within the interlayer region, whereas the band located at 1022 cm^−1^ can be associated with the prohibited stretching mode of the nitrate group [36]. The bands at 882 and 832 cm^−1^ can be assigned to the C–O vibrational mode of carbonate anions [37]. The bands around 764 cm^−1^, 639 cm^−1^, and 462 cm^−1^ are attributed to the vibrations of Zn–O [21]. For the host matrix ZHA, the broad band at 3440 cm^−1^, just like in ZHN, can be assigned to the O–H stretching vibration mode. The bands around 1560 and 1400 cm^−1^ are associated with COO^−^ asymmetric and symmetric vibrational modes, respectively. The difference in the ν_a_(COO^−^) and ν_s_(COO^−^) (Δν_a-s_ = 160 cm^−1^) stretching frequency indicates an ionic mode in the coordination of the carboxylate group to the layered structure, according to the description given by Nara et al. [38].

The bands at 1334 and 1049 cm^−1^ can be associated with the vibration modes of the methyl group (CH_3_) due to deformation and rocking, respectively [39]. The weak band at 831 cm^−1^ is attributed to the CO_3_^2−^ scissoring vibration mode, and the bands at lower wavenumbers are assigned to the Zn–OH vibrations. For both intercalated compounds, the bands associated with the starting anions, located in the interlayer distances (1378 and 1560–1400 cm^−1^) of the host matrices, were not evident. These changes also demonstrate the immobilization process of the caffeate anions in the interlayer region. For ZHN–CA, the broad band centered at 3440 cm^−1^ is associated with the OH vibration mode of the layers and water molecules located in the interlayer space [19], whereas, for ZHA–CA, in this region, the bands at 3431 and 3230 cm^−1^ are assigned to the stretching vibration mode of the OH group of the aromatic ring of the caffeic acid molecule [40,41]. These results agree with the thermal analysis, where the loss mass due to the water molecules was lower in ZHA–CA than for ZHN–CA in the temperature range 30 °C–150 °C. The bands observed in the range from 1598 to 1421 cm^−1^ are attributed to the stretching vibration mode of the C–C group. The bands at 1643 and 838 cm^−1^ are related to the C=O group vibration mode. The band at around 1267 cm^−1^ corresponds to the stretching vibration mode of the C–OH group [41,42].

The bands in the range from 1116 to 796 cm^−1^ are associated with the different vibration modes of the CH group, with the higher wavenumber due to the in-plane bending and the lower wavenumber due to out-of-plane bending vibration [42]. The bands at 723 and 671 cm^−1^ are assigned to the aromatic ring out-of-plane bending mode of the C–C group and in-plane bending mode of the C=O group, respectively. The band around 592 cm^−1^ can be assigned to out-of-plane bending O–H vibration, whereas the band at 551 cm^−1^ corresponds to the aromatic ring in-plane bending mode of the C–C group [43]. The vibrations at lower wavenumbers can be attributed to the Zn–O stretching vibration [36]. These results are in accordance with the XRD patterns for the immobilization process of caffeate anions for both layered inorganic systems, ZHN and ZHA [19].

### 3.6. Scanning Electron Microscopy (SEM)

**Figure 6:**
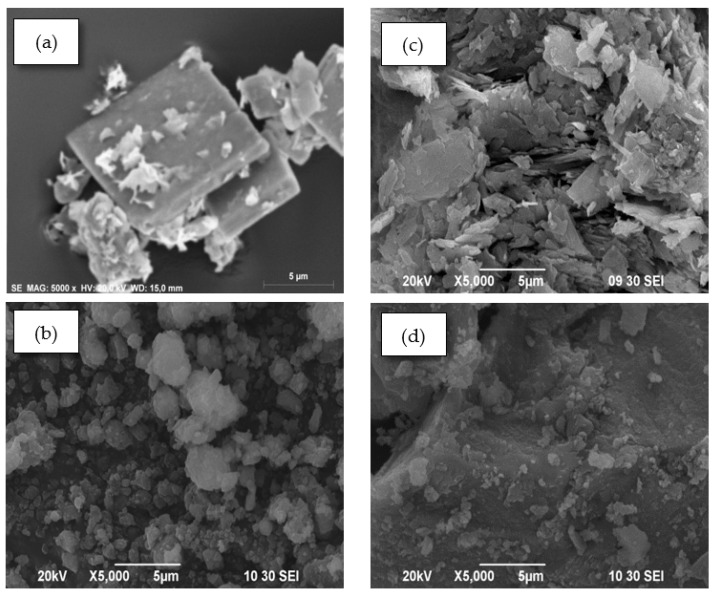
SEM images of (**a**) ZHN host matrix, (**b**) ZHN–CA, (**c**) ZHA, and (**d**) ZHA–CA.

The SEM images for ZHN and ZHA, as well as those for the hybrid materials, ZHN–CA and ZHA–CA, are shown in Figure 6. ZHN (Figure 6a) exhibited well-defined crystals with the characteristic plate-like morphology of layered materials. The morphology of the intercalated compound ZHN–CA (Figure 6b) showed agglomerated particles with a heterogeneous distribution. For the ZHA host matrix, a non-uniform sheet and flake-like structure were observed (Figure 6c). However, ZHA–CA exhibited a dense structure with a smoother surface and small particles on the surface (Figure 6d). These results show clear differences in the morphology between the inorganic host matrices and the hybrid materials, which can affect the general properties of the materials.

### 3.7. X-Ray Photoelectron Spectroscopy (XPS)

**Figure 7:**
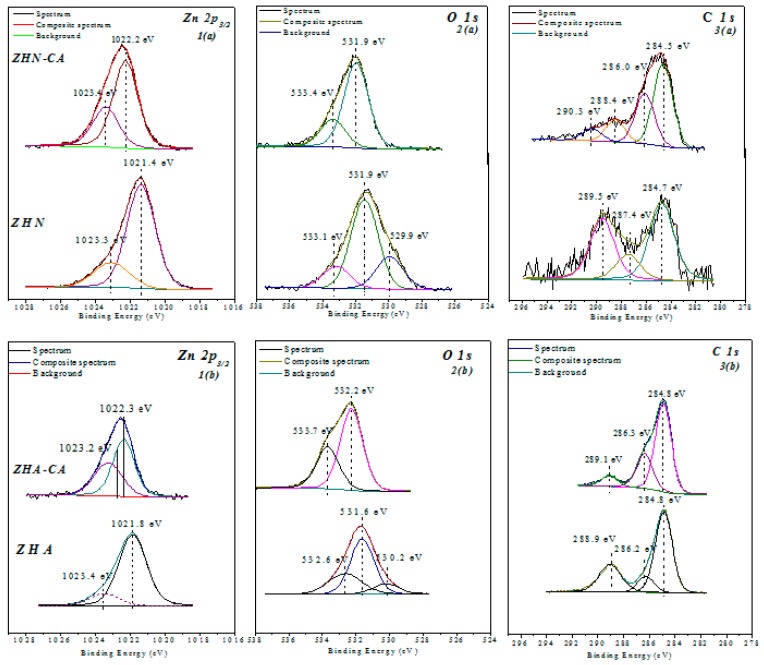
High-resolution XPS of chemical state (**1**) Zn 2*p*_3/2_, (**2**) O 1*s*, and (**3**) C 1*s* for (**a**) ZHN and ZHN–CA, and (**b**) ZHA and ZHA–CA.

The high-resolution XPS spectra of the chemical states of Zn, C, and O are shown in Figure 7a,b for the inorganic host matrices and hybrid materials. For the ZHN host matrix, the Zn 2*p*_3/2_ core level spectrum exhibited two contributions with binding energies at 1021.4 and 1023.1 eV. These contributions are related to the structural characteristics of the layered host matrix, where the Zn^2+^ atoms have tetrahedral and octahedral coordination in the Zn_5_(OH)_8_(NO_3_)_2_∙2H_2_O structure [14,22]. The binding energy values for O 1*s* were 529.9, 531.4, and 533.1 eV, which correspond to CO_3_^2−^, –OH, and NO_3_^−^/H_2_O, respectively. The presence of carbonate ions is due to the high affinity of the layers with compatible chemical species such as CO_2_ [21]. Based on the peak fitting, the intense peak corresponds to the contribution of Zn(OH)_2_, which forms the layers. In contrast, the peak at higher binding energy is related to the nitrate anions that act as counterions, and adsorbed water molecules occupying the interlayer spaces of the layered structure. For the C 1*s* core-level spectrum, three contributions were detected with energies of 284.7, 287.4, and 289.5 eV, associated with C–H, C=O, and CO_3_^2−^, respectively. The lower binding energy value is assigned to adventitious carbon, which is usually found on the surface of most samples exposed to air, whereas the two higher binding energy values are associated with the presence of the carbonate ion.

For the intercalated compound ZHN–CA, the C 1*s* core-level spectrum showed four contributions after the peak fitting. The band at 284.5 eV is assigned to C–H, due to the aromatic ring of the caffeate anion, as well as to the adventitious carbon. The chemical state of C 1*s* at 286.0 eV is attributed to the carboxylate group (COO^−^), which participated in the covalent bonding with the Zn^2+^ tetrahedral units of the layers to form the hybrid compound. The band around 288.4 eV corresponds to the phenolic group (C–OH) confirming the caffeic acid molecule, and the weak band at a higher binding energy, 290.3 eV, is assigned to the presence of the carbonate anion in the intercalation compound. The O 1*s* core level for the hybrid compound, ZHN–CA, exhibited two bands at 531.9 eV and 533.4 eV. The band at the lower binding energy corresponds to the Zn(OH)_2_ which forms to the layers, and the band at the higher binding energy can be associated with the carboxylate group (COO^−^) of the organic molecule. For the hybrid material, two chemical states for Zn 2*p*_3/2_ were identified at 1022.2 and 1023.4 eV, which are related to the different chemical environments of the Zn^2+^ atom in the octahedral and tetrahedral units of the layered structure.

In the case of the ZHA host matrix, the C 1*s* core-level spectrum showed three bands with binding energy values at 284.8, 286.2, and 288.9 eV. The band at the lower binding energy value is assigned to the C–H associated with the presence of the methyl group of the acetate molecule and the adventitious carbon. The band around 286.2 eV is related to C=O bonds, and the band at the higher binding energy value is also assigned to the presence of the carboxylate group of the acetate anion [22]. These results agree with the FT-IR analysis previously described. The O 1*s* core-level spectrum exhibited three contributions. The band around at 530.2 eV is assigned to the presence of carbon species such as CO_2_. The strong band at 531.6 eV corresponds to the Zn(OH)_2_ forming the layers, and the band at the higher binding energy value (532.6 eV) is related to the carboxylate group (COO^−^) of the acetate anions. From the Zn 2*p*_3/2_ core level, two peaks were identified, one of them corresponds to Zn^2+^ with octahedral coordination to the hydroxyl groups at 1021.8 eV, and the other is a weak band associated with the tetrahedral coordination of the Zn^2+^ atom.

For the hybrid material ZHA–CA, the C 1*s* core-level spectrum exhibited only three contributions at 284.8, 286.3, and 289.1 eV. The component related to carbon species at higher binding energy was not found like for ZHN–CA. However, for the O 1*s* and Zn 2*p*_3/2_ core-level spectra, similar contributions were identified after the peak fitting of this hybrid material. The results of XPS are in accordance with those of XRD and FT-IR analysis, which could confirm the immobilization process of the organic molecules in both starting layered structures, since, although the starting materials were different, the hybrid compounds gave rise to a single arrangement of the caffeate anions in both layered structures. The displacement of the binding energy values for the different core levels, C1*s*, O 1*s*, and Zn 2*p*_3/2_, between the host matrices and the hybrid compounds, is related to the change in chemical environment after the interaction, through H-bonding, between the water molecules of the tetrahedral units of the layers and the carboxylate group of caffeate anions. For both inorganic host matrices, ZHN and ZHA, the binding energy values for the tetrahedral coordinated Zn^2+^ ions were similar. Nevertheless, the change in the intensity of this contribution can be related to the structural variation of the layered host, since, for ZHA, the amount of water in crystallization may vary, depending on the synthesis method [20], thereby affecting its availability and the possibility of interaction with guest organic molecules.

### 3.8. Controlled Release

**Figure 8:**
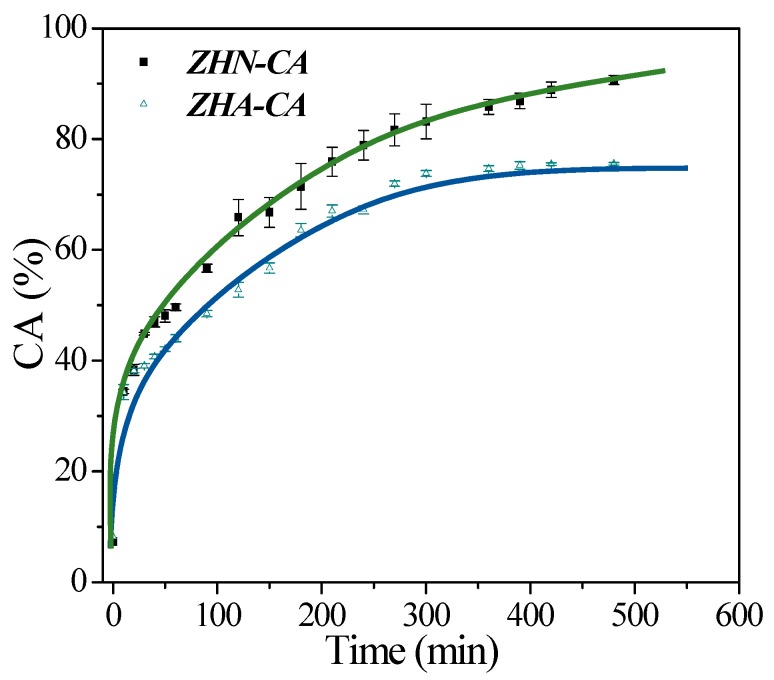
Release profiles for CA from ZHN–CA and ZHA–CA in buffer solutions at pH 7.1. Solid lines are a guide for the behavior of the releasing profile and have no physical significance. The curves correspond to the average of three experimental measurements for each point.

The CA release profiles of ZHN–CA and ZHA–CA in pH 7.1 buffer solution are given in Figure 8. For ZHN–CA, a faster release of CA was reached in comparison to the hybrid compound ZHA–CA. The CA released amount was about ~80% in 300 min for ZHN–CA, whereas only ~66% was released for ZHA–CA. These results reveal differences in the interactions between the organic anions and the layers of the two host matrices, ZHA and ZHN. As a result, there was a stronger coordination bonding between carboxylate groups of the caffeate anions with the ZHA than that with the ZHN, in concordance with the analysis of interfacial characterization made in the previous section (Section 3.2). Therefore, the release process was slower and more restricted than that for ZHN–AC. For ZHA–CA, the release behavior achieved an equilibrium after 420 min with ~74%, whereas, for ZHN–CA, 480 min were necessary with ~95% release. The results indicate that the hybrid compounds have potential use as controlled release systems.

### 3.9. Kinetic Analysis

**Figure 9:**
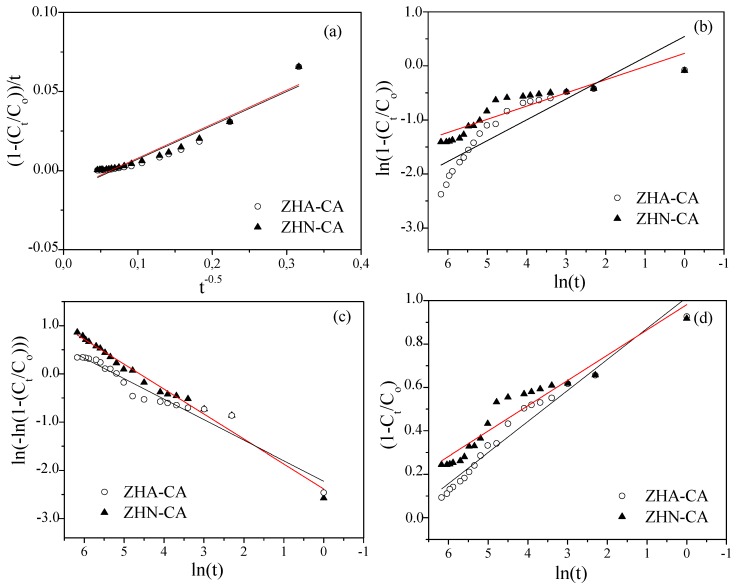
Plots of kinetic models to the experimental release of CA anions from ZHN–CA and ZHA–CA hybrid materials: (**a**) parabolic, (**b**) Freundlich, (**c**) Avrami-Erofe’ev, and (**d**) Elovich models.

To explore the kinetics of the release process of caffeate anions from the hybrid compounds, four models were evaluated. The release data were fitted by the parabolic, Freundlich, Elovich, and Avrami-Erofe’ev models, and the fitted plots are displayed in Figure 9. The equations of these models and the *R*^2^ coefficients from the fitting are presented in Table 2. It was found that the release data fitted well when using the Elovich and Avrami-Erofe’ev models for both hybrid compounds. The model provided better fitting of the data for ZHN–CA with the correlation coefficient being higher than for ZHA–CA. Based on these results, the release processes for the hybrid compounds were mainly governed by the surface diffusion [16,42], and the better release behavior for ZHN–CA can be related to the chemical composition, structural characteristics, and morphology of this hybrid compound compared to ZHA–CA.

## 4. Conclusions

Caffeic acid was successfully immobilized into the ZHN and ZHA host matrices. Variations of the chemical composition of the hybrid materials were estimated from the elemental analysis, and the values of zeta potential evidenced differences in the surface charge of the starting layered host matrices, which affected the resulting layered structures of the hybrid materials.

The basal spacing of ZHN–CA was 10.20 Å and, for ZHA-CA, the *d*-value was 10.37 Å, indicating that the caffeate anions were arranged, with a tilt angle of 70°, along the *z*-axis in the interlayer space of the ZHN and ZHA structures. The strong interaction between the carboxylate groups of the caffeic acid molecule and the Zn^2+^ of the tetrahedral units produced the substitution of the water molecules occupying the apex of the tetrahedron, giving rise to the arrangement of the CA anions in the interlayer region.

The immobilization of caffeate anions in both host matrices was confirmed by FT-IR analysis and XPS analysis, although the differences in chemical environment of the starting layered structures defined the superficial characteristics and morphology of the hybrid compounds. The total mass loss during the heat treatment was higher for the intercalation compound ZHN–CA (34.68%) than for ZHA–CA (37.09%) due to the difference in the amount of adsorbed water and the water in crystallization molecules of the host matrices. An enhancement of 65 °C of the thermal stability was observed for ZHN–CA due to the retardation of the dehydroxylation process. The release profiles indicated a lower release of CA for ZHA–CA, which is related to the stronger interaction between the layers and the guest anions. The Elovich and Avrami-Erofe’ev models described the release process, through a surface diffusion mechanism, for both hybrid compounds. These results indicate that ZHN–CA and ZHA–CA have potential for use as controlled release formulations of organic molecules, with antioxidant properties.

## Figures and Tables

**Table 1:** Chemical composition and calculated formula of the hybrid materials.

Hybrid Material	Calculated Formula	Elemental Content Percentages Observed (Calculated) (wt.%)			
		Zn	C	N	H
**ZHN**	Zn_5_(OH)_8_(NO_3_)_1.6_(CO_3_)_0.2_·1.7H_2_O *	54.5 (54.1)	0.4 (0.4)	3.6 (3.7)	1.9 (1.9)
**ZHA**	Zn_5_(OH)_7.5_(C_2_H_3_O_2_)_2.5_∙3H_2_O	50.0 (49.8)	9.3 (9.1)	-	3.2 (3.2)
**ZHN–CA**	Zn_5_(OH)_8_(C_9_H_7_O_4_)_2_	38.7 (39.8)	27.5 (26.3)	-	2.4 (2.7)
**ZHA–CA**	Zn_5_(OH)_7.5_(C_9_H_7_O_4_)_2.5_∙H_2_O	38.1 (35.5)	29.3 (25.2)	-	3.0 (2.9)

* Chemical composition of the zinc hydroxide nitrate reported in previous work [21].

**Table 2:** Equations of kinetic models for release analysis and *R*^2^ coefficients of fitted results.

Model	Equation	*R* ^2^	
		ZHN–CA	ZHA–CA
**Parabolic**	(1 − *C*_t_/*C*_0_)/*t* = *k*_d_(*t* − *t*_0_)^−0.5^ + *a*	0.9293	0.9139
**Freundlich**	ln(1 − *C*_t_/*C*_0_) = ln(*k*_d_) + *a*ln((*t* − *t*_0_))	0.8378	0.7837
**Elovich**	1 − *C*_t_/*C*_0_ = *a*ln((*t* − *t*_0_)) + *b*	0.9554	0.9206
**Avrami-Erofe’ev**	ln(−ln(*C*_t_/*C*_0_)) = nln*k*_d_ + nln(*t* − *t*_0_)	0.9754	0.9494

*C*_0_ represents the amount of CA anions in ZHN and ZHA at *t* = 0, *C*_t_ is the amount of CA anions in ZHN and ZHA at time *t*, and *k*_d_ is the rate of release. *a*, *b*, and *n* are constants. The *R*^2^ coefficients were obtained from three replicas of the response taken for each time level by using the statistical package XLSTAT-2019 [43].
